# Urinary candidate biomarker discovery in a rat unilateral ureteral obstruction model

**DOI:** 10.1038/srep09314

**Published:** 2015-03-20

**Authors:** Yuan Yuan, Fanshuang Zhang, Jianqiang Wu, Chen Shao, Youhe Gao

**Affiliations:** 1 Department of Pathophysiology, National Key Laboratory of Medical Molecular Biology, Institute of Basic Medical Sciences Chinese Academy of Medical Sciences, School of Basic Medicine Peking Union Medical College, Beijing, 100005, China; 2 Department of Biochemistry and Molecular Biology, Beijing Normal University, Gene Engineering and Biotechnology Beijing Key Laboratory, Beijing, 100875, China

## Abstract

Urine has the potential to become a better source of biomarkers. Urinary proteins are affected by many factors; therefore, differentiating between the variables associated with any particular pathophysiological condition in clinical samples is challenging. To circumvent these problems, simpler systems, such as animal models, should be used to establish a direct relationship between disease progression and urine changes. In this study, a unilateral ureteral obstruction (UUO) model was used to observe tubular injury and the eventual development of renal fibrosis, as well as to identify differential urinary proteins in this process. Urine samples were collected from the residuary ureter linked to the kidney at 1 and 3 weeks after UUO. Five hundred proteins were identified and quantified by LC-MS/MS, out of which 7 and 19 significantly changed in the UUO 1- and 3-week groups, respectively, compared with the sham-operation group. Validation by western blot showed increased levels of Alpha-actinin-1 and Moesin in the UUO 1-week group, indicating that they may serve as candidate biomarkers of renal tubular injury, and significantly increased levels of Vimentin, Annexin A1 and Clusterin in the UUO 3-week group, indicating that they may serve as candidate biomarkers of interstitial fibrosis.

Urine has the potential to become a better biological source for disease biomarker discovery, particularly for renal diseases, because this fluid accumulates changes in the body and is the direct excreta of the kidney^1^,^2^. Changes in urinary proteins are caused by various factors; therefore, identifying the specific variables associated with a particular pathophysiological condition in clinical samples remains challenging. To circumvent these issues, simpler systems such as animal models should be used to establish a direct relationship between disease progression and corresponding changes in urine^3^.

Renal fibrosis is regarded as the final common pathway for most forms of progressive renal disease and involves glomerular sclerosis and/or interstitial fibrosis^4^. Most renal disorders lead to renal fibrosis; therefore, there is great interest in identifying biomarkers for early diagnosis or therapeutic monitoring. In obstructive nephropathy, interstitial fibrosis is caused by tubular injury and the proliferation of interstitial fibroblasts, and this process is considered the most serious pathological change in end-stage renal disease^4^,^5^,^6^. Many pre-clinical studies have provided extensive insights into the common pathways of fibrosis; however, few studies have focused on identifying urinary biomarkers involved in this pathological process^7^.

The unilateral ureteral obstruction (UUO) model was developed to identify useful biomarkers in different stages of obstructive nephropathy. In the 1970s, a rabbit UUO model demonstrated fibroblast proliferation and transformation in the renal interstitium^8^. Since this finding, UUO animal models have been considered the classic models of obstructive nephropathy because UUO can be easily manipulated by changing the time, severity and duration of the experimental setup. This animal model is used to elucidate the pathogenesis of obstructive nephropathy and the pathological mechanisms mediating renal fibrosis^9^,^10^. Furthermore, this model mimics different stages of obstructive nephropathy and possibly leads to the infiltration of inflammatory cells, tubular expansion and apoptosis, myofibroblast accumulation and differentiation (from tubular epithelium, pericytes and perivascular fibroblasts^11^), extracellular matrix deposition, and tubular atrophy^12^. These different pathological features appear rapidly and are highly reproducible. In this study, urine samples were collected from the residuary ureter linked to the kidney following UUO, providing samples similar to the specimens obtained from patients with obstructive nephropathy.

Several studies have applied proteomics analyses to characterize candidate renal tissue markers in animal models^13^,^14^; however, whether these molecules are released into bodily fluids and can serve as non-invasive markers remains unclear. Other studies have illustrated urine markers using samples from human for ureteropelvic junction obstruction and congenital bilateral hydronephrosis^14^,^15^. Urinary proteins may be affected by multiple physiological and pathological factors, especially in human samples, and these proteins are affected by several common medicines^16^. Therefore, sample sizes need to be very large to distinguish differences, even between individuals.

This study was designed to identify urinary biomarkers related to renal tubular injury and interstitial fibrosis in the early (1 week) and late (3 week) stages of a rat UUO model using urinary proteomic profiling.

## Methods

### Experimental animals and surgical procedures

Specific pathogen-free male Sprague-Dawley rats weighing 180–200 g were purchased from the Institute of Laboratory Animal Science, Chinese Academy of Medical Science & Peking Union Medical College. The animal experiments were reviewed and approved by the Institute of Basic Medical Sciences Animal Ethics Committee, Peking Union Medical College (Approved ID: ACUC-A02-2014-008). All animals were maintained with a standard laboratory diet under controlled indoor temperature (22 ± 1°C) and humidity (65–70%) conditions. The study was performed strictly according to the guidelines developed by the Institutional Animal Care and Use Committee of Peking Union Medical College. All efforts were made to minimize suffering.

Animals were anesthetized with 2% pelltobarbitalum natricum (40 mg/kg body weight), and the left ureter was exposed and separated through a flank incision. In the rats undergoing UUO, the left ureter was ligated with 4-0 silk at two points and then severed between the ligatures, whereas in the sham-operated rats, the left ureter was left undisturbed. The rats were studied 1 week and 3 weeks after the operation (n = 6 rat per group). Urine was collected from the residuary ureter linked to the kidney by ureter catheterization for 3 hours before the rats were sacrificed. In first 0.5 hours, the urine collected had accumulated during induction of UUO, and in the final 2.5 hours, newly generated urine from the kidney injured by UUO was collected. Only the urine collected during the final 2.5 hours was used in the following experiments. Urine from the sham-operated rats was collected from the left ureter in the same manner as for the UUO rats. Just before the kidneys were removed, 2–3 ml of blood was collected from the abdominal aorta in a heparinized tube and centrifuged at 3000 × *g* for 20 min at 4°C to obtain serum. The left kidney was then harvested. One half of the kidney was fixed, and the other half was used for protein extraction.

### Renal histopathology

For histopathology, the left kidneys of all rats were harvested 1 or 3 weeks after operation. One half of each kidney was fixed with 10% phosphate-buffered formalin and embedded in paraffin. Then, the paraffin sections (4 μm thick) were stained with hematoxylin and eosin (HE) to reveal histopathological lesions. Kidney fibrosis was evaluated by Masson's trichrome staining^17^.

### Laboratory biochemical analysis

Urine and serum were analyzed in the clinical laboratory of Peking Union Medical College Hospital. The urine protein and creatinine, serum creatinine, blood urea nitrogen, serum HDL-C and LDL-C concentrations were measured. Then, after 24 hours, the urine protein-to-creatinine ratio (Up/Ucr) was calculated to evaluate proteinuria level in place of 24-hour urinary protein^18^.

### Urinary protein sample preparation

Urine was centrifuged at 2,000 × *g* for 15 min. After removing the cell debris, the supernatant was centrifuged at 12,000 × *g* for 15 min at 4°C. Urinary proteins were extracted from the individual urine samples by acetone precipitation, and the pellets were re-suspended with lysis buffer as previously described^19^. Protein concentrations in each urine sample were measured using the Bradford method.

### Proteome analysis

The proteins were digested with trypsin (Trypsin Gold, Mass Spec Grade, Promega, Fitchburg, WI, USA) using filter-aided sample preparation methods^20^. A total of 200 μg protein was loaded onto 10-kD filter devices (Pall, Port Washington, NY, USA). UA (8 M urea in 0.1 M Tris-HCl, pH 8.5) and NH_4_HCO_3_ (50 mM) were added successively to wash the proteins, and the tube was centrifuged at 14000 g for 20 min at 18°C. The proteins were denatured by incubation with 5 mM dithiothreitol (DTT) at 50°C for 1 h, and then alkylated with 50 mM iodoacetamide (IAA) in the dark for 45 min. After washing twice with UA and four times with NH_4_HCO_3_, the proteins were re-dissolved in NH_4_HCO_3_ and digested with trypsin at an enzyme to protein ratio of 1:100 at 37°C overnight. The tryptic peptide mixtures were collected by centrifugation. The filtrates were desalted using Oasis HLB cartridges (Waters, Milford, MA) and then dried by vacuum evaporation (Thermo Fisher Scientific, Bremen, Germany).

The digested peptides were acidified and re-dissolved in 0.1% formic acid, then loaded onto a reversed-phase microcapillary column using a Waters UPLC system. The peptides were separated on a 10-cm fused silica column. The peptides were eluted over a gradient of 5%–28% buffer B (0.1% formic acid, 99.9% ACN; flow rate, 0.3 μL/min) for 100 min. MS data were acquired using the ABSCIEX Triple-TOF5600 mass spectrometer system (Framingham, MA, US). Three out of six animals were randomly chosen. Each sample was analyzed three times. The MS/MS data were processed using Mascot software (version 2.4.1, Matrix Science, London, UK) and searched against the Swiss-Prot rat database (05/03/2013; containing 9,354 sequences). For peptide identification, the fragment ion mass tolerance was set to 0.05 Da, and the parent ion tolerance was set to 0.05 Da. The search allowed for two missed cleavage sites in the trypsin digestion. The carbamidomethylation of cysteines was considered a fixed modification, and both the oxidation of methionine and the deamidation of asparagines were considered variable modifications. The Mascot results were filtered and validated by Scaffold (version 4.0.1, Proteome Software Inc., Portland, OR, USA). Peptide identifications were accepted if they were detected with ≥90.0% probability and an FDR less than 0.1% by the Scaffold local FDR algorithm. Protein identifications were accepted if they were detected with ≥95.0% probability and contained at least 2 identified peptides^21^.

### Spectral counting analysis for differential proteins

Spectral counting was used to compare protein abundance between different experimental groups according to procedure described previously^22^,^23^,^24^. The proteins with spectral counts that differed between the sham-operation group and the UUO group above internal experimental variation were defined as differential proteins. To increase reliability and because the total spectral counts in the UUO groups were lower than in the sham-operation group, we chose differential proteins whose spectral counts were more than 2-fold higher in the UUO 1-w or 3-w group than in the sham-operation group.

### Immunohistochemical analysis

The paraffin-embedded sections from individual samples were permeabilized with 0.2% Triton and blocked with 5% bovine serum albumin (BSA) in 0.1 M phosphate-buffered saline (PBS) for 30 minutes to reduce nonspecific binding. Then, the samples were incubated with primary antibodies against ACTN1 (1:200, AJ1029a, ABGENT, San Diego, CA, USA), Clusterin (1:200, AF1255a, ABGENT, San Diego, CA, USA), Moesin (1:200, ab52490, Abcam, Cambridge, MA, USA), Vimentin (1:200, ab9547, Abcam, Cambridge, MA, USA) and Annexin A1 (1:200, ab138512, Abcam, Cambridge, MA, USA), followed by a biotinylated secondary antibody (PV-9000, Beijing ZSGB-Bio, Beijing, China). The samples were incubated with the IgG κ light chain (1:200, M0809-1, Hangzhou HuaAn Biotechnology Company, Hangzhou, China) of similar concentration as the primary antibody controls, followed by the same biotinylated secondary antibody (PV-9000, Beijing ZSGB-Bio, Beijing, China). Immunoreactivity was visualized with DAB, and brown staining was considered a positive result.

### Western blot analysis

Five proteins that were differentially expressed in urine and kidney tissue samples and related to kidney diseases or organ fibrosis were verified by western blot. A total of 60 μg urine protein and 60 μg tissue protein from individual samples were separated on a 10% SDS-PAGE and electrotransferred to PVDF membranes. Membranes were blocked with 5% non-fat milk and incubated overnight at 4°C with primary antibodies [anti-ACTN1 (1:500, AJ1029a), anti-Clusterin (1:500, AF1255a), ABGENT; anti-Moesin (1:500, ab52490), anti-Vimentin (1:500, ab92547) and anti-Annexin A1 (1:500, ab138512), Abcam] followed by horseradish peroxidase-conjugated secondary antibody (1:5000, 074-1506, Kirkegard and Perry Laboratories, USA). Target bands were visualized using enhanced chemiluminescence (ECL) reagents. ECL results were scanned and analyzed using an ImageQuant 400TM Imager (GE Healthcare Life Sciences, Piscataway, NJ, USA), and the intensity of each protein band was quantified using ImageJ analysis software (National Institutes of Health, Bethesda, MD, USA).

### Statistical analysis

Comparisons between independent groups were conducted using one-way ANOVA followed by post hoc analysis with the least significant difference (LSD) test or Dunnett's T3 test. Group differences resulting in P-values of less than 0.05 were considered statistically significant. Statistical analysis was performed using the Statistical Package for Social Studies (SPSS) version 16.0.

## Results

### Histopathological changes in the kidneys of UUO rats

**Figure 1:**
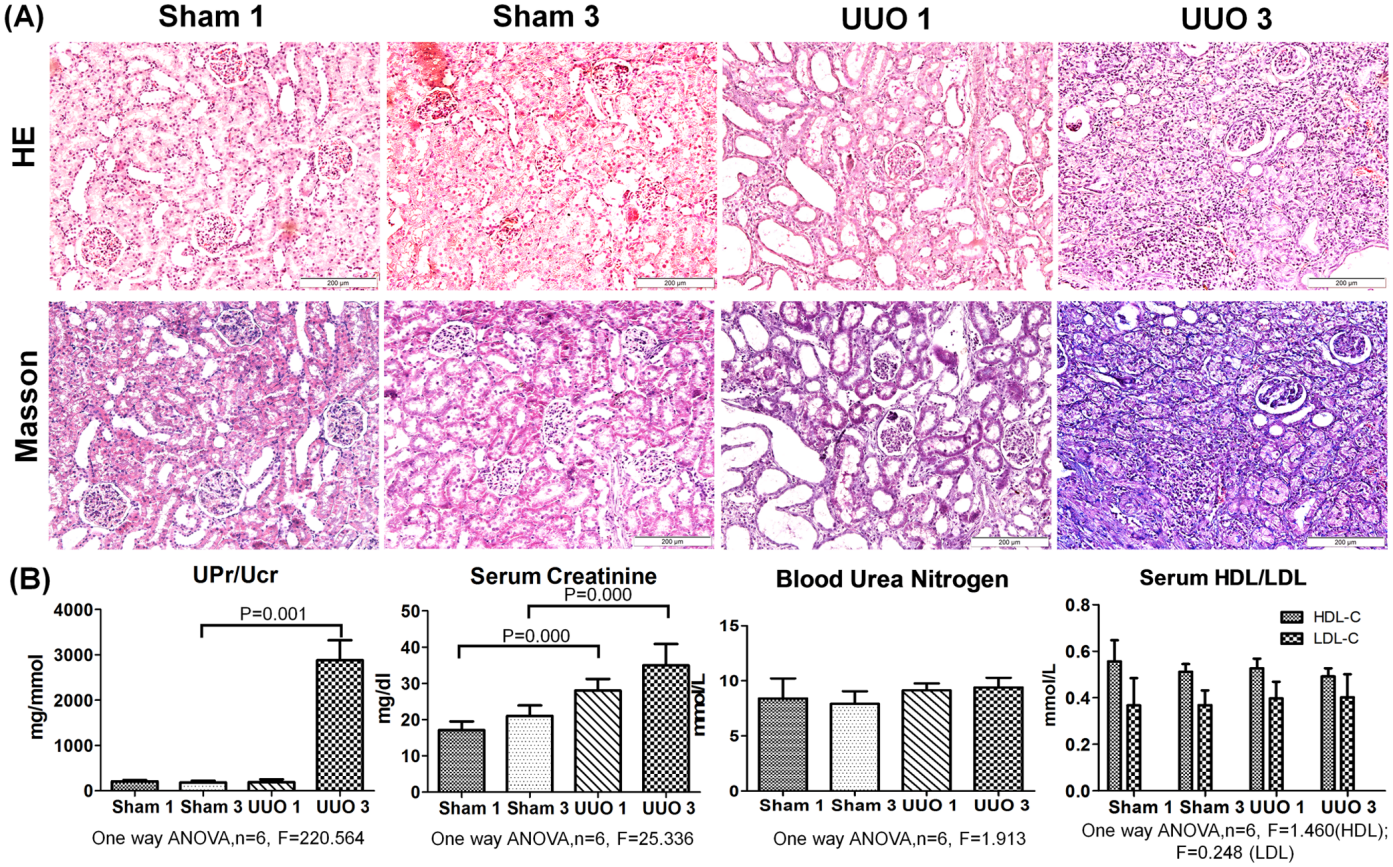
Pathological morphologies and the biochemical indicators of urine/serum in sham-operated and UUO rats. (A) HE and Masson's trichrome staining of Sprague-Dawley rat kidneys at 1 and 3 weeks after UUO. (B) Up/Ucr ratio, serum creatinine, urea nitrogen and HDL/LDL. The data represent the mean ± SD. Sham 1: sham-operation 1-week group; Sham 3: sham-operation 3-week group; UUO 1: UUO 1-week group; UUO 3: UUO 3-week group.

The HE staining of kidneys obtained from sham-operated and UUO rats are shown in Figure 1. In the UUO 1-week group, the glomerular morphology was almost normal compared with the sham-operation group, whereas the tubules were expanded with mildly broadened interstitial space and inflammatory cell infiltration in the interstitium. In the UUO 3-week group, most of the Bowman's capsules were broadened, several of the glomeruli were destroyed, several tubules displayed atrophy with a thickened basement membrane, and the interstitium was notably broader and displayed significant inflammatory cell infiltration in the interstitial space. Masson's trichrome staining revealed only limited renal interstitial fibrosis in the UUO 1-week group, whereas this phenomenon was much more extensive in the UUO 3-week group. All of the changes in the renal tissue suggested that the UUO model was successful and that typical fibrosis appeared 3 weeks after UUO (Figure 1).

### Up/Ucr ratios and serum creatinine levels increased in the UUO model

The Up/Ucr ratio was 15.61-fold higher in the UUO 3-week group than in the sham-operation 3-week group. Serum creatinine levels increased 1.25-fold in the UUO 1-week group relative to the sham-operation 1-week group and 1.29-fold in the UUO 3-week group relative to the sham-operated 3-week group (Figure 1). Mean levels of blood serum urea nitrogen, HDL-C and LDL-C were generally higher in the UUO group than in the sham-operated group, but not significantly.

### Urinary proteome changes identified by LC-MS/MS

The same proteomic analyses were used to analyze the protein composition of urine from rats in the sham-operated group, the UUO 1-week group and the UUO 3-week group. For each of these groups, nine samples (three samples each taken from three rats per group) were analyzed by UPLC coupled with Triple-TOF 5600 MS. Each sample was analyzed three times to provide technical replicates (Table 1). For the total difference in spectra between the sham-operated group and the UUO group, spectral counting was used to perform semi-quantitative analysis [16–18]. In this study, the abundance of each given protein in a sample was estimated by the mean spectral count of proteins that existed in all 3 replicates.

In total, 500 proteins were identified by scaffold integration in the sham-operated and UUO groups. Relative to the sham-operated group, we found 65 differentially expressed proteins in the UUO 1-week group, and 74 in the UUO 3-week group. Fifty-one overlaps existed between these two sets of differential proteins. To be conservative, all of the differential proteins met the following criteria: 1) only proteins with at least two unique peptides were included; 2) the variation trend in all three animals was consistent, and the fold-change was >2 in at least one animal; and 3) the spectral counting in each animal in the UUO group was >5. Overall, 7 significantly changed proteins (Table 2) were observed between the sham-operated and UUO 1-week groups, and 19 significantly changed proteins (Table 3) between the sham-operated and UUO 3-week groups. Two overlaps were observed between these sets of differentially expressed proteins. We selected 5 proteins related to kidney disease or organ fibrosis that had specific antibodies available for validation in urine and kidney tissue. These proteins were Alpha-actinin-1 (fold change = 14.00), Annexin A1 (fold change = 5.96), Clusterin (fold change = 2.90), Moesin (fold change = 18.00) and Vimentin (fold change = 6.00).

### Validation of differentially expressed proteins by western blot and immunohistochemistry

**Figure 2:**
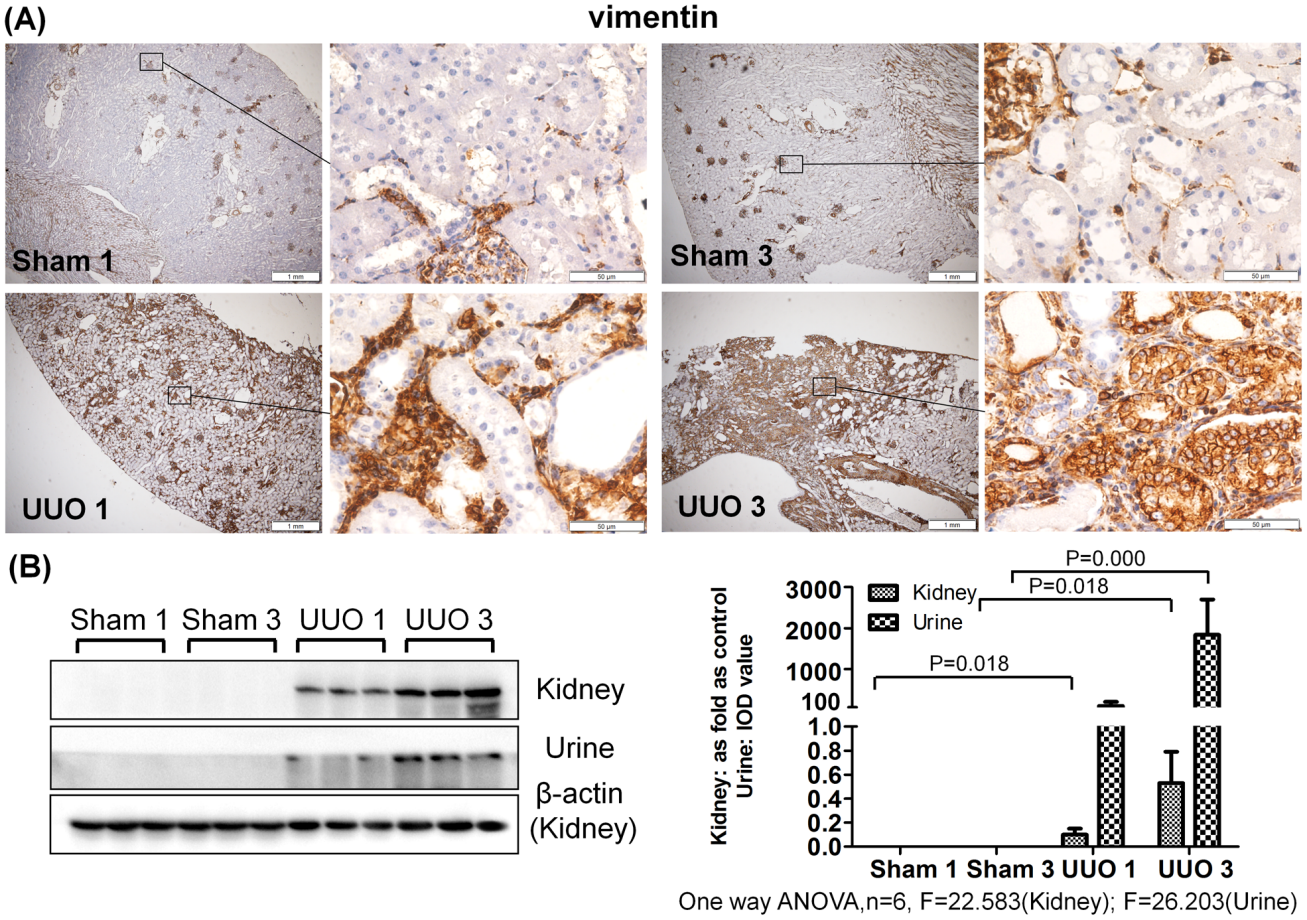
Vimentin accumulated in urine and obstructed kidneys after UUO. (A) The expression and distribution of Vimentin in kidney analyzed by immunohistochemistry. (B) The expression of Vimentin in kidney and urine measured by western blot. The histogram shows quantitative representations of Vimentin in the kidney (normalized to β-actin) and urine (IOD value) obtained from densitometric analysis by IPP 6.0 software. The data represent mean ± SD. Sham 1: sham-operation 1-week group; Sham 3: sham-operation 3-week group; UUO 1: UUO 1-week group; UUO 3: UUO 3-week group.

**Figure 3:**
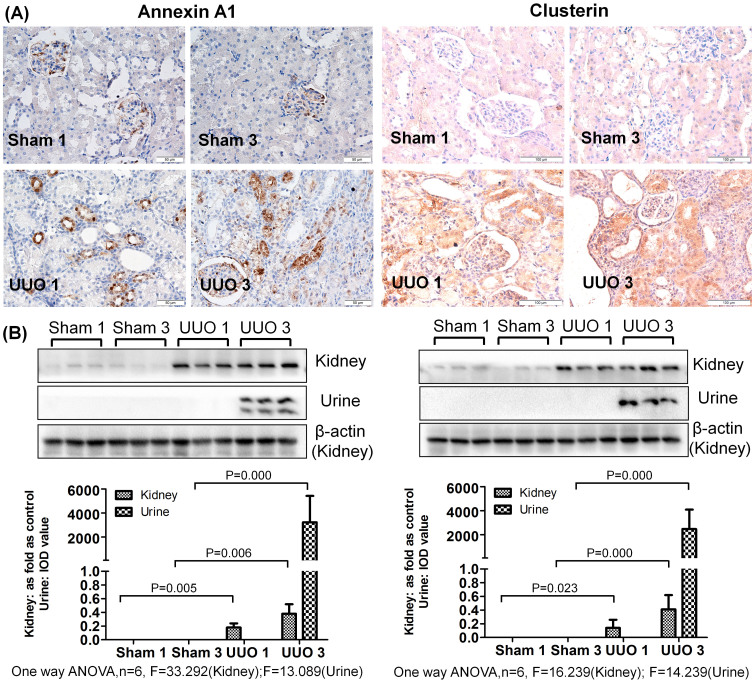
Levels of Annexin A1 and Clusterin were increased in urine and obstructed kidneys after UUO. (A) The expression and distribution of Annexin A1 and Clusterin in kidney analyzed by immunohistochemistry. (B) The expression of Annexin A1 and Clusterin in kidney and urine measured by western blot. The histograms show the quantitative representation of Annexin A1 and Clusterin in the kidney (normalized to β-actin) and urine (IOD value) obtained from densitometric analysis by IPP 6.0 software. The data represent mean ± SD. Sham 1: sham-operation 1-week group; Sham 3: sham-operation 3-week group; UUO 1: UUO 1-week group; UUO 3: UUO 3-week group.

As shown in Figure 2,3,4, a western blot of urine proteins revealed no apparent change in the level of Vimentin, Annexin A1 or Clusterin in the UUO 1-week group but a significant increase in the UUO 3-week group. These results were consistent with the mass spectrometry results. However, a western blot of kidney tissue proteins showed increased levels of Vimentin, Annexin A1 and Clusterin after 1 week of UUO. The levels of Alpha-actinin-1 and Moesin in urine were increased 1 week after unilateral ureteral obstruction, a result consistent with western blot results of Alpha-actinin-1 and Moesin in kidney tissue.

Immunohistochemistry revealed positive staining of Vimentin in interstitial cells around tubules in the UUO 1-week group. The same protein was also evidently expressed in damaged tubular epithelial cells and interstitial fibrotic areas in the UUO 3-week group. In the sham-operated groups (both 1-week and 3-week), Vimentin staining was only positive in the kidney glomerulus and not in the interstitial space or kidney tubules. Furthermore, little to no expression of Annexin A1, Clusterin, Alpha-actinin-1 and Moesin was observed in the sham-operated groups. Interestingly, positive staining for these proteins was observed in tubular epithelial cells of the UUO model, with higher expression in the UUO 3-week group than in the UUO 1-week group. The controls with IgG κ light chain in all groups showed no positive staining (Supplementary Figure 1).

## Discussion

Urine is an excellent source of biomarkers because this fluid accumulates changes, a hallmark that is the most fundamental property of biomarkers^3^. Urinary proteins are affected by multiple physiological and pathological factors, including age, gender, diet, exercise, and medication^16^. However, some properties of urine have historically caused difficulties in pinpointing factors associated with any specific pathophysiological condition in clinical samples. To minimize these challenges, animal models can be utilized. Obstruction of the ureter can lead to complex renal injury and insufficiency^25^ as well as protein changes in both serum and urine. Although increased Up/Ucr ratio and serum creatinine levels indicate renal injury, these markers cannot distinguish between specific nephropathies. In this study, UUO, a widely used model of kidney disease associated with progressive tubulointerstitial damage, was utilized to mimic the progression of obstructive nephropathy. This approach enabled the identification of several differentially expressed proteins by urinary proteomic profiling. In humans, obstructive nephropathy always involves incomplete obstruction. Therefore, the urine collected from the residuary ureter linked to the kidney after UUO may mimic urine from patients with obstructive nephropathy.

The common pathological features of UUO are parenchymal loss (tubular injury) and progressive interstitial fibrosis. In the majority of previous UUO studies, samples were taken at an early stage (7 days) and a later stage when parenchymal damage was evident (14 days or later)^26^. Here, we profiled urinary proteomes at UUO 1-week and 3-week. Levels of several of the differential proteins identified between the sham-operated group and the UUO group, such as Aminopeptidase N, Vimentin, and Lumican, had previously been reported to be higher in damaged kidney tissue in obstructive nephropathy studies^27^,^28^,^29^. Although several of the proteins identified were not related to obstructive nephropathy, they were associated with other renal diseases and served as potential markers of various pathologies. For example, the significant up-regulation of glycogen phosphorylase has been reported in diabetic nephropathies^30^. Cathepsin D promotes the fibrogenic potential of hepatic stellate cells^31^ and is increased in the saliva of cystic fibrosis patients^32^. Most of the differentially expressed proteins had not been detected in urine from the UUO model. In this study, the differential proteins in urine were considered to play an important role in the regulation of obstructive nephropathy and possibly serve as key markers during the progression of this disease. The proteins that changed after 1 week of UUO may serve as candidate renal tubular injury biomarkers, whereas proteins changed after 3 weeks of UUO may reflect renal interstitial fibrosis.

The validation of 5 differentially expressed proteins further confirmed the results of our proteome analysis. Moreover, the validation results reflected both the similarities and differences between kidney tissue and urine. Both this study and others found significant evidence that the positive expression of Vimentin in the early stage (1-week) is indicative of the accretion of interstitial cells, whereas in the later stage (3-week), an epithelial–mesenchymal transition in tubules and the formation of irreversible interstitial fibrosis is suggested^28^. Therefore, Vimentin may be a potential urinary marker for renal fibrosis. The increased levels and abnormal expression of Annexin A1, Clusterin, Alpha-actinin-1 and Moesin were observed in tubular epithelial cells in the UUO model and were correlated with the pathological stage. Annexin A1 is a potential biomarker in renal cell carcinoma and lupus nephritis^33^,^34^. In another study of UUO, the up-regulation of Annexin A1 was reported 2 and 8 days following UUO in the renal parenchyma^35^. Alpha-actinin-1 is an essential component of the glomerular filtration barrier and has been implicated in the pathogenesis of familial focal segmental glomerulosclerosis, nephrotic syndrome, IgA nephropathy, focal segmental glomerulosclerosis and minimal change disease^34^. Clusterin participates in the process of renal aging and acute kidney injury^36^,^37^. The differential expression and location of these proteins provide new clues to help explore the mechanisms of tubular injury and collateral fibrosis. Alpha-actinin-1 and Moesin detected after 1 week of UUO may be used as potential biomarkers of tubular injury, whereas Annexin A1, Clusterin and Vimentin may be the candidate markers of renal fibrosis.

Several differentially expressed proteins appear in many different nephropathies, which suggests that these proteins may be the common markers of multiple diseases that share similar pathological changes^38^. In a previous study, a rat model of adriamycin (ADR)-induced nephropathy was used to find candidate biomarkers for focal segmental glomerulosclerosis^39^. Distinct differential proteins were present in the ADR-induced nephropathy and UUO models. Serum albumin, Serotransferrin and Kallikrein-1 were detected in both studies but displayed opposite trends. In the UUO 1-week group, the glomerular morphology was almost normal, whereas obvious glomerular injury appeared in the UUO 3-week group. Kallikrein-1 decreased 3 weeks after UUO and in the ADR-induced nephropathy model, which indicates that Kallikrein-1 is a candidate marker of glomerular injury. The expression profile of differentially expressed proteins in these studies illustrates that specific pathological conditions have their own characteristic changes and biomarker combinations.

Although high-abundance proteins are easier to identify as clinical markers, more detailed and comprehensive proteomic analysis detects additional specific changes. In future studies, other animal models and higher-resolution time points may provide better candidate biomarkers. In addition, a large number of clinical samples should be used to verify specific proteins or protein patterns as clinically applicable biomarkers of obstructive nephropathy.

## Author Contributions

Y.G. and Y.Y. conceived and designed the experiment. Y.Y. performed the experiments, analyzed data and wrote the manuscript. Y.Y. performed the LC–MS/MS analysis; F.Z., J.W. and C.S. analyzed data. Y.G. helped to revise the manuscript. All authors read and approved the final manuscript.

## Supplementary Material

Supplementary Information Supplementary Information

## Figures and Tables

**Figure 4:**
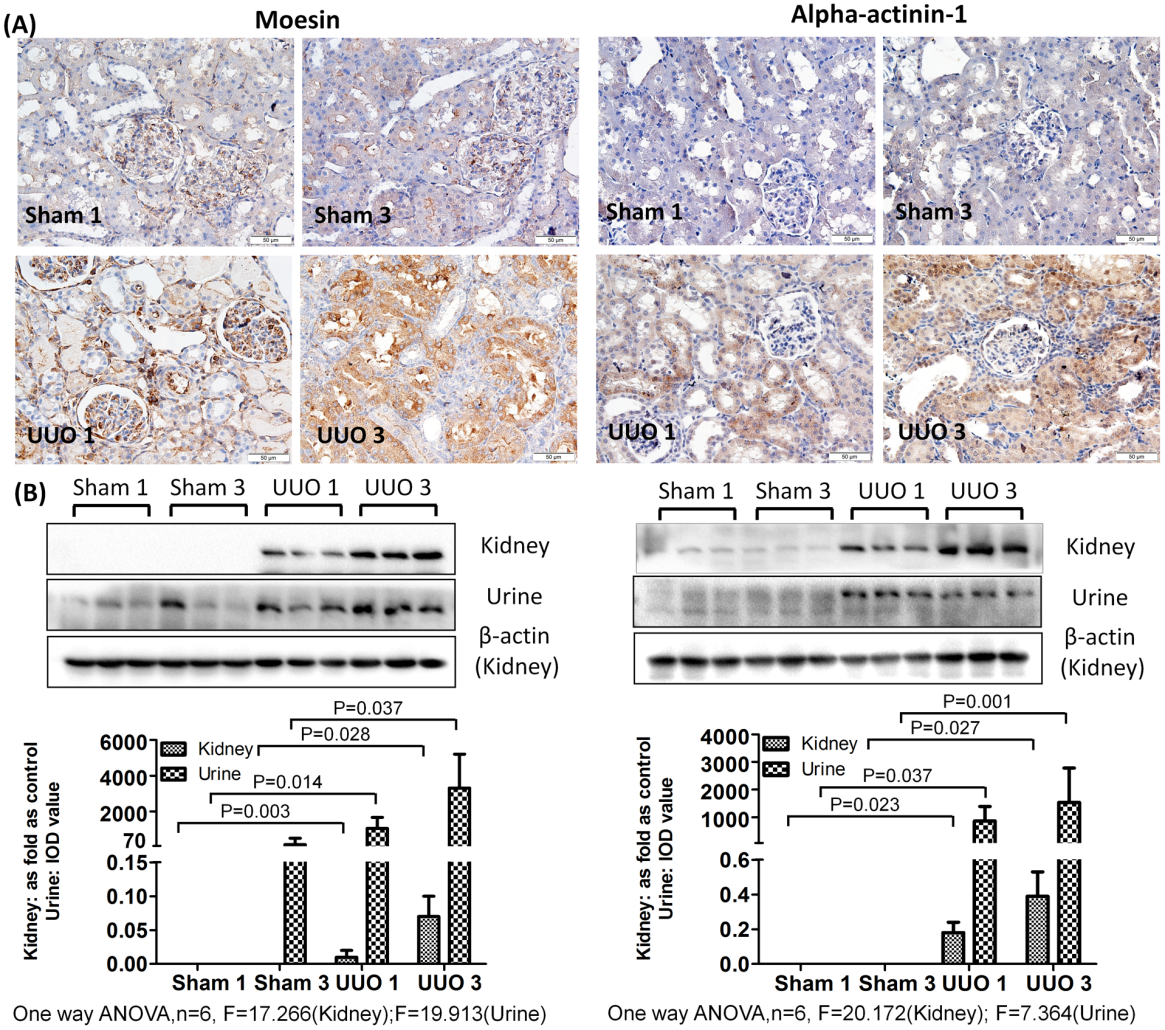
Moesin and Alpha-actinin-1 are increased in urine and the obstructed kidneys after UUO. (A) The expression and distributions of Moesin and Alpha-actinin in the kidney analyzed by immunohistochemistry. (B) The expression of Moesin and Alpha–actinin-1 in kidney and urine measured by western blot. The histograms show quantitative representations of Moesin and Alpha-actinin-1 in the kidney (normalized to β-actin) and urine (IOD value) obtained from densitometric analysis by IPP 6.0 software. The data represent mean ± SD. Sham 1: sham-operation 1-week group; Sham 3: sham-operation 3-week group; UUO 1: UUO 1-week group; UUO 3: UUO 3-week group.

**Table 1:** Proteins identified from urine samples

Group	Biological repeat	Technical repeat	Identified proteins
Sham-operationgroup	Sham 1	1	305
		2	324
		3	337
	Sham 2	1	254
		2	253
		3	265
	Sham 3	1	269
		2	266
		3	278
UUO 1-week group	UUO 1-1	1	233
		2	240
		3	249
	UUO 1-2	1	191
		2	194
		3	197
	UUO 1-3	1	271
		2	275
		3	265
UUO 3-week group	UUO 3-1	1	222
		2	216
		3	236
	UUO 3-2	1	275
		2	273
		3	275
	UUO 3-3	1	275
		2	289
		3	288

**Table 2:** Proteins displaying significant changes between the sham-operated and UUO 1-week groups

					Spectral counts						
Protein name	Accession Number	Fold- change	P value	F value	Sham1	Sham2	Sham3	UUO1-1	UUO1-2	UUO1-3	Disease with up-regulation
Aminopeptidase N	AMPN_RAT	2.94	0.002	26.925	15	13	7	41	25	37	obstructive nephropathy^27^
Cathepsin D	CATD_RAT	4.98	0.01	9.528	8	39	10	106	51	127	bladder cancer^40^
Galectin-3-binding protein	LG3BP_RAT	5.20	0.01	8.212	10	9	1	41	20	43	breast cancer^41^
Glycogen phosphorylase, muscle form	PYGM_RAT	32.00	0.001	35.848	1	0	0	12	12	8	acute uremia^42^
Intraflagellar transport protein 172 homolog	IF172_RAT	29.00	0.005	9.349	0	0	1	14	10	5	none
Protein S100-A9	S10A9_RAT	12.50	0.004	20.447	0	2	0	7	6	12	type 1 diabetes mellitus^43^
Solute carrier family 12 member 7	S12A7_RAT	24.00	0.008	7.879	1	0	0	6	11	7	Gitelman syndrome^44^

**Table 3:** Proteins displaying significant changes between the sham-operated and the UUO 3-week groups

					Spectral counts						
Protein name	Accession Number	Fold- change	P value	F value	Sham1	Sham2	Sham3	UUO3-1	UUO3-2	UUO3-3	Disease with up-regulation
Alpha-actinin-1	ACTN1_RAT	14.00	0	78.11	0	2	0	8	10	10	rheumatoid arthritis^45^
Alpha-actinin-4	ACTN4_RAT	2.90	0.017	12.02	4	3	3	6	14	9	IgA nephropathy^46^
Annexin A1	ANXA1_RAT	5.96	0.001	25.10	2	11	10	47	47	43	Mesangial proliferative glomerulonephritis^47^
Cluster of Histone H1.4	H14_RAT	20.00	0.001	22.47	0	2	0	13	18	9	none
Cluster of Vimentin	VIME_RAT^23^	6.00	0	183.77	2	5	2	17	19	18	Obstructive nephropathy^28^
Clusterin	CLUS_RAT	2.90	0.006	10.69	32	31	35	79	117	87	melamine- and cyanuric acid-induced kidney injury^48^
Complement component C9	CO9_RAT	2.16	0.015	6.76	18	40	37	67	68	70	obstructive nephropathy^49^
Extracellular superoxide dismutase [Cu-Zn]	SODE_RAT	9.00	0.005	10.85	0	2	0	6	7	5	ischemia-reperfusion-induced acute kidney injury^50^
Glycogen phosphorylase, muscle form	PYGM_RAT	38.00	0	35.85	1	0	0	10	14	14	acute uremia^42^
Golgi resident protein GCP60	GCP60_RAT	29.00	0	34.68	0	1	0	12	10	7	none
Ig gamma-1 chain C region	IGHG1_RAT	2.43	0.007	8.60	9	11	8	29	18	21	pancreatic cancer^51^
Lumican	LUM_RAT	3.40	0.003	12.69	5	3	2	12	9	13	obstructive nephropathy^29^
Moesin	MOES_RAT	18.00	0.01	7.12	2	0	0	9	18	9	Diabetic nephropathy^52^
Periaxin	PRAX_RAT	38.00	0.002	13.57	1	0	0	9	14	15	none
Protein S100-A8	S10A8_RAT	9.50	0.002	13.94	1	3	0	16	14	8	type 1 diabetes mellitus^43^
Protein S100-A9	S10A9_RAT	16.50	0.001	20.45	0	2	0	10	12	11	type 1 diabetes mellitus^43^
Serine protease inhibitor A3N	SPA3N_RAT	2.83	0.006	8.46	32	92	67	181	196	163	none
Transaldolase	TALDO_RAT	6.00	0.034	27.32	2	3	0	10	9	11	diabetic nephropathy^53^
