# Supplementary material for: Urinary candidate biomarker discovery in a rat unilateral ureteral obstruction model
Source: Sci Rep. 2015 Mar 20;5:9314. doi: 10.1038/srep09314 (PMC4366765; doi:10.1038/srep09314)
Supplement: Supplementary Information [file srep09314-s1.doc]

**Supplementary information**

**Urinary candidate biomarker discovery in a rat unilateral ureteral obstruction model**

**Yuan Yuana, FanshuangZhanga, JianqiangWua, Chen Shaoa, Youhe Gao * a, b**

**a. Department of Pathophysiology, National Key Laboratory of Medical Molecular Biology, Institute of Basic Medical Sciences Chinese Academy of Medical Sciences School of Basic Medicine Peking Union Medical College/Peking Union Medical College, Beijing 100005, China**

**b.Department of Biochemistry and Molecular Biology, Beijing Normal University, Gene Engineering and Biotechnology Beijing Key Laboratory, Beijing, 100875, P. R. of China**

**All correspondence forYouheGao:**

**Requests for offprints should be addressed to Prof. YouheGao, Department of Biochemistry and Molecular Biology, Beijing Normal University, Gene Engineering and Biotechnology Beijing Key Laboratory, Beijing, 100875, P. R. of China**

**Phone:** **+86-10- 6915-6407**

**Email: youhegao@163.com**

**Supplementary Figure legends**

**Supplementary Figure. 1. Immunohistochemical analysis of controls**


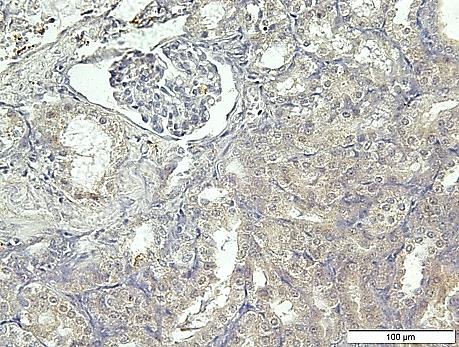


**Sham 1**


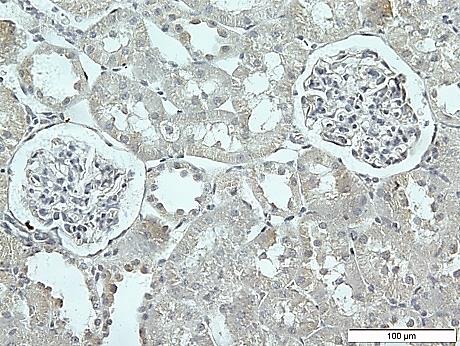


**Sham 3**


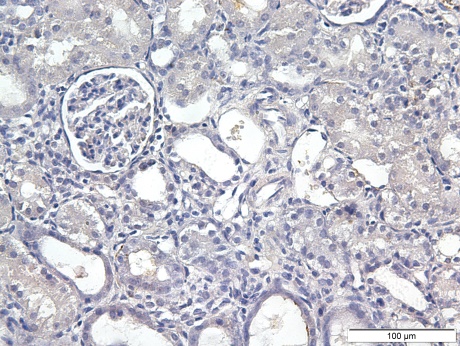


**UUO1**


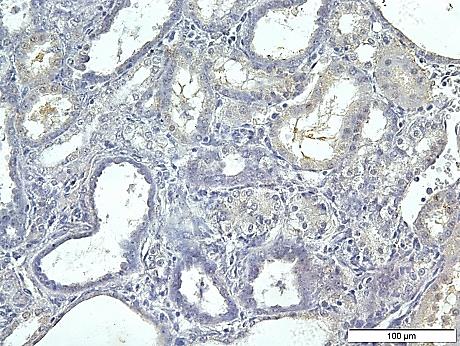


**UUO 3**

**Supplementary Figure. 2. Full length blots of Figure 2-4**

Marker

Sham 1

Sham 3

UUO 1

UUO 3

Marker

**
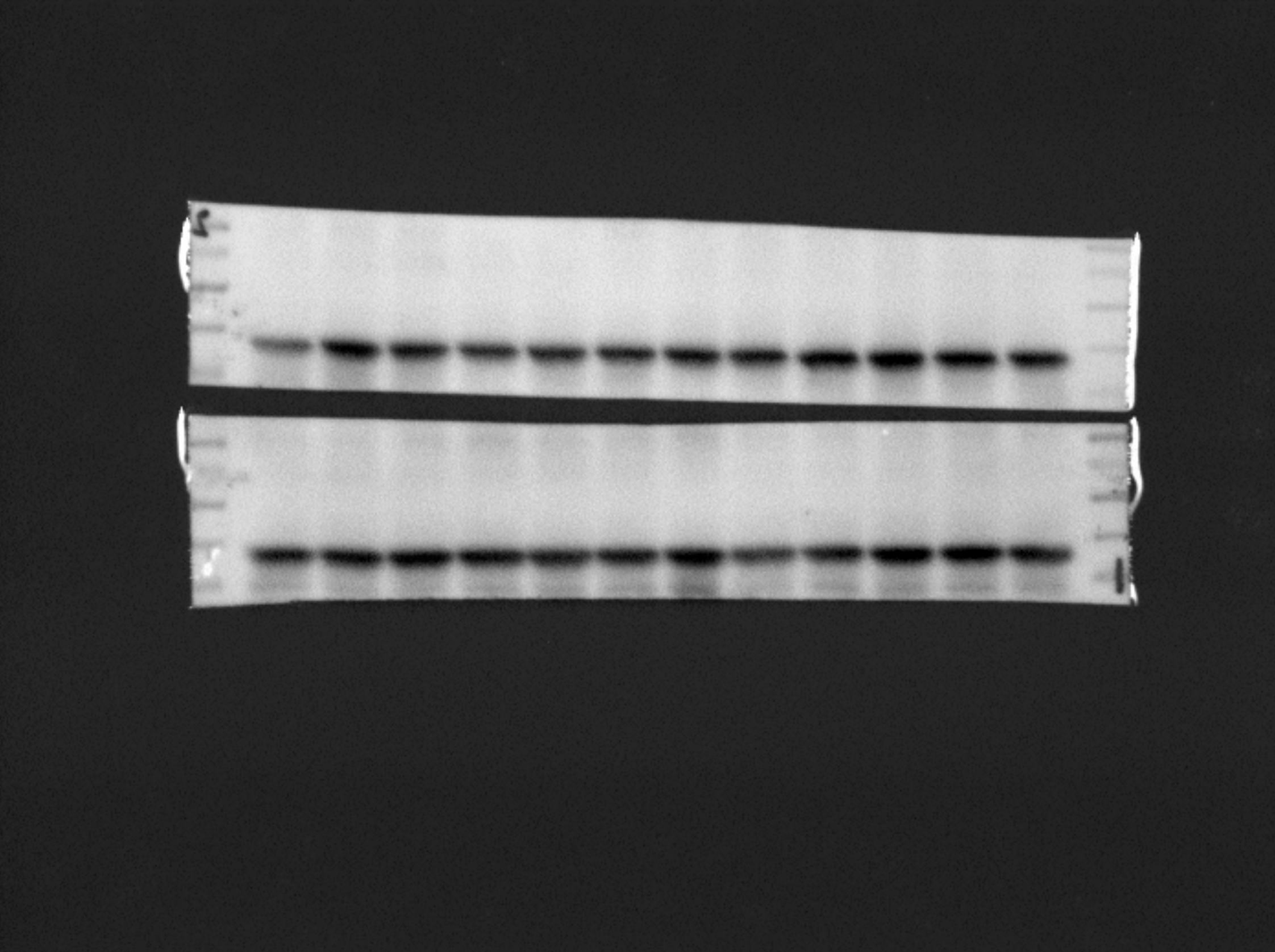
**

1

2

3

1

2

3

1

2

3

1

2

3

40KD-

4

5

6

4

5

6

4

5

6

4

5

6

**β-actin**

Marker

Sham 1

Sham 3

UUO 1

UUO 3

1

2

3

1

2

3

1

2

3

1

2

3

4

5

6

4

5

6

4

5

6

4

5

6

70KD-

55KD-

Marker

**Vimentin**


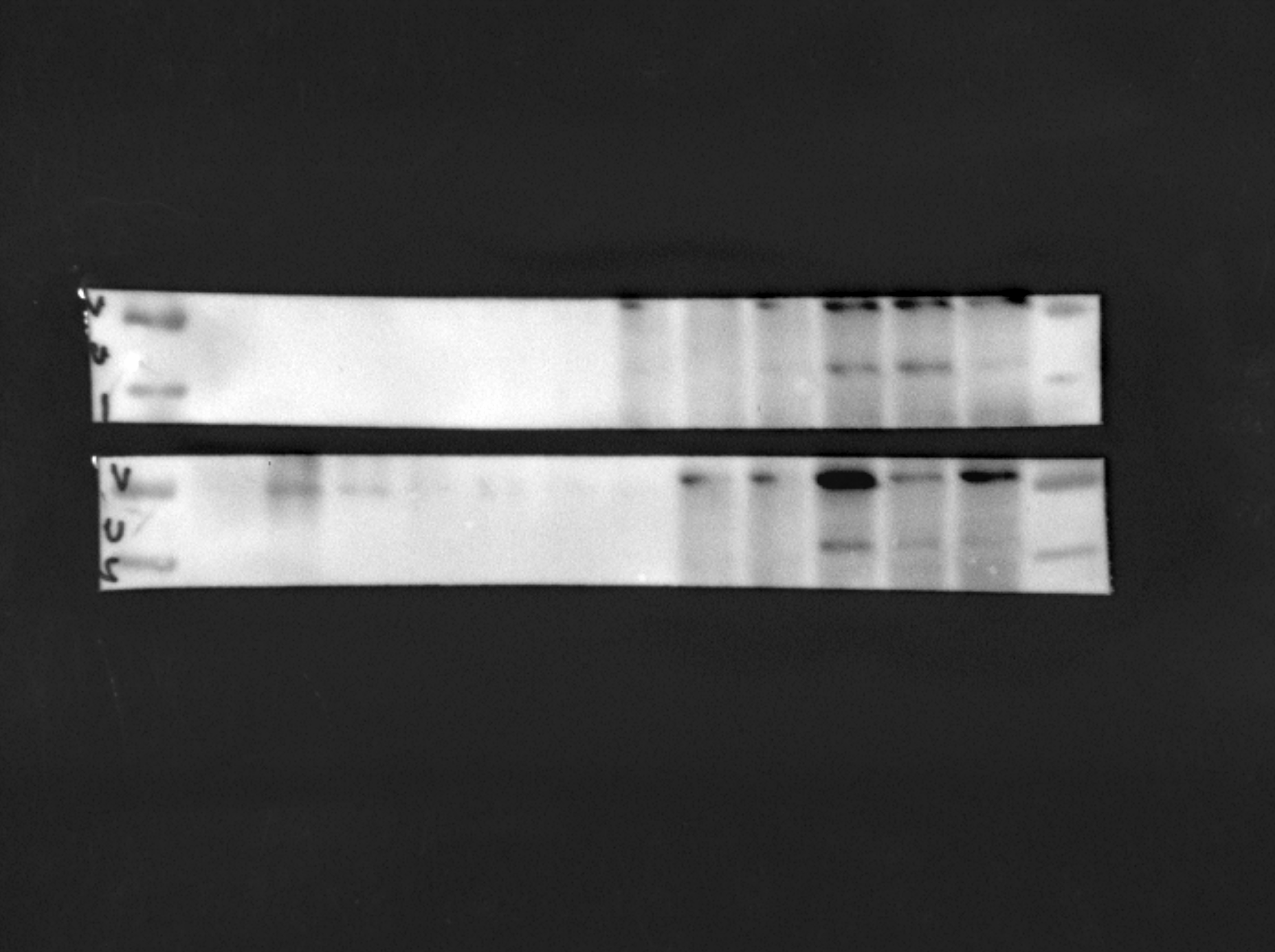


Marker

Sham 1

Sham 3

UUO 1

UUO 3

1

2

3

1

2

3

1

2

3

1

2

3

4

5

6

4

5

6

4

5

6

4

5

6

55KD-

40KD-

Marker

**Urine**

**Kidney**

**
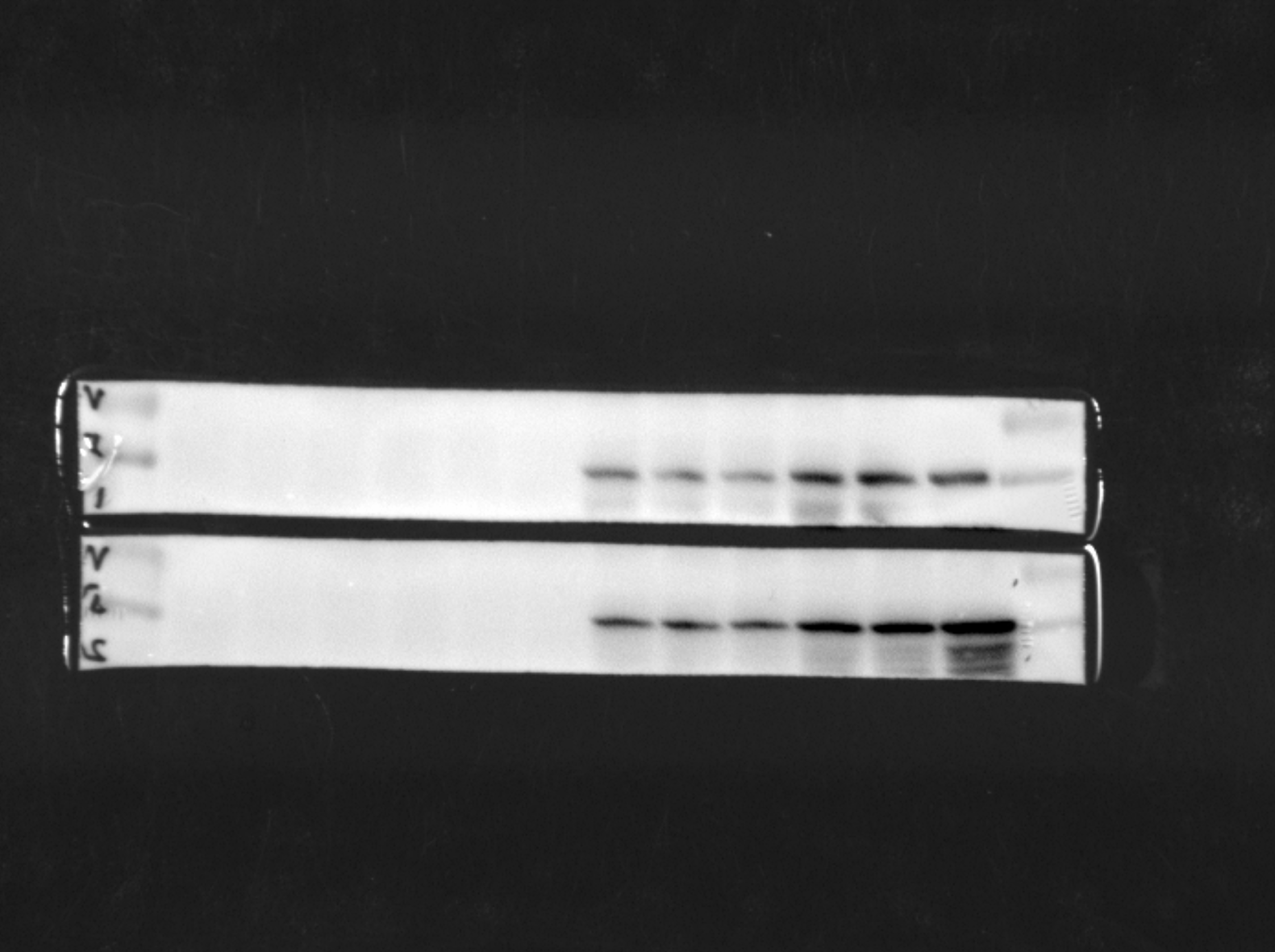
**

**
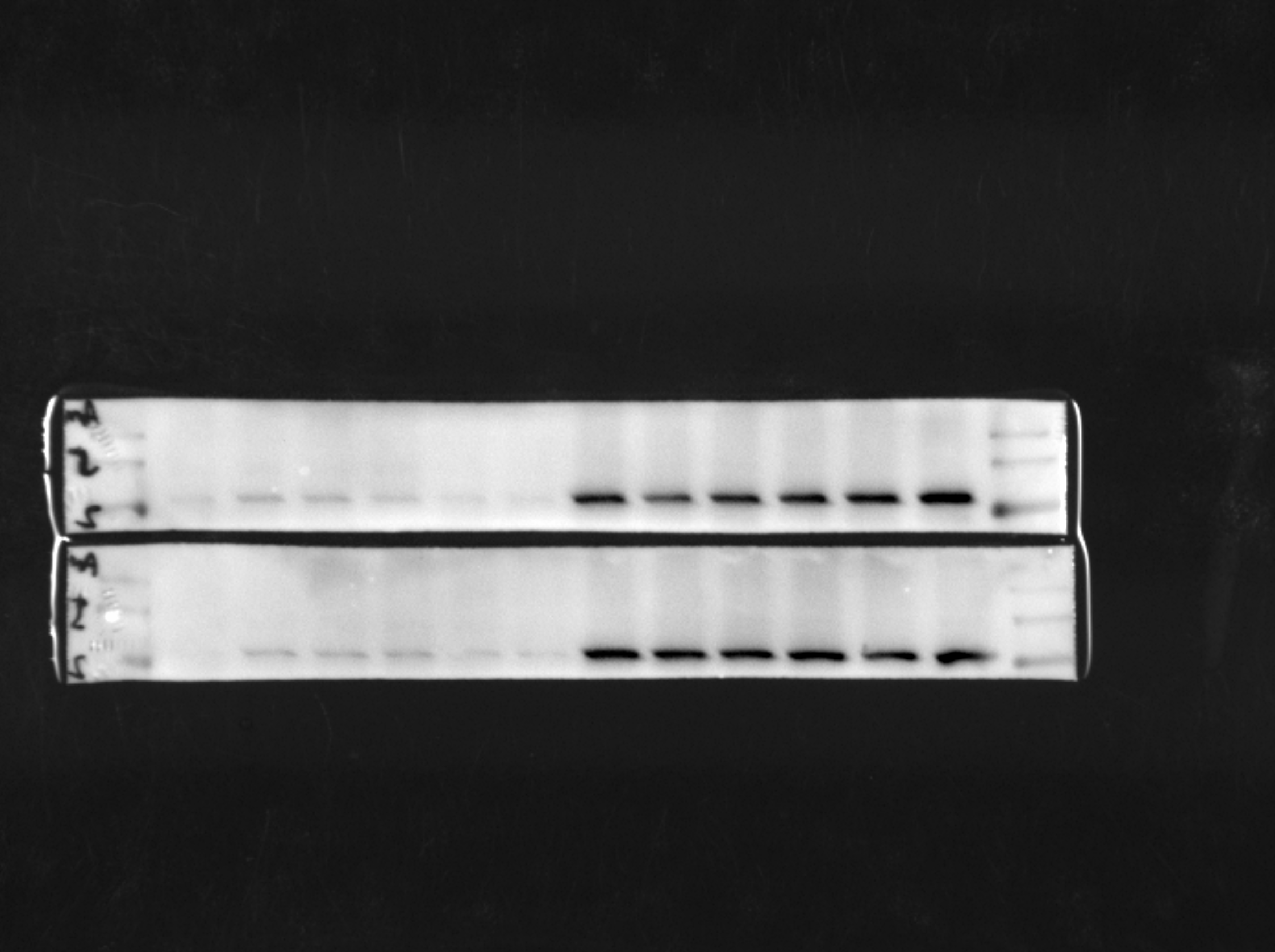

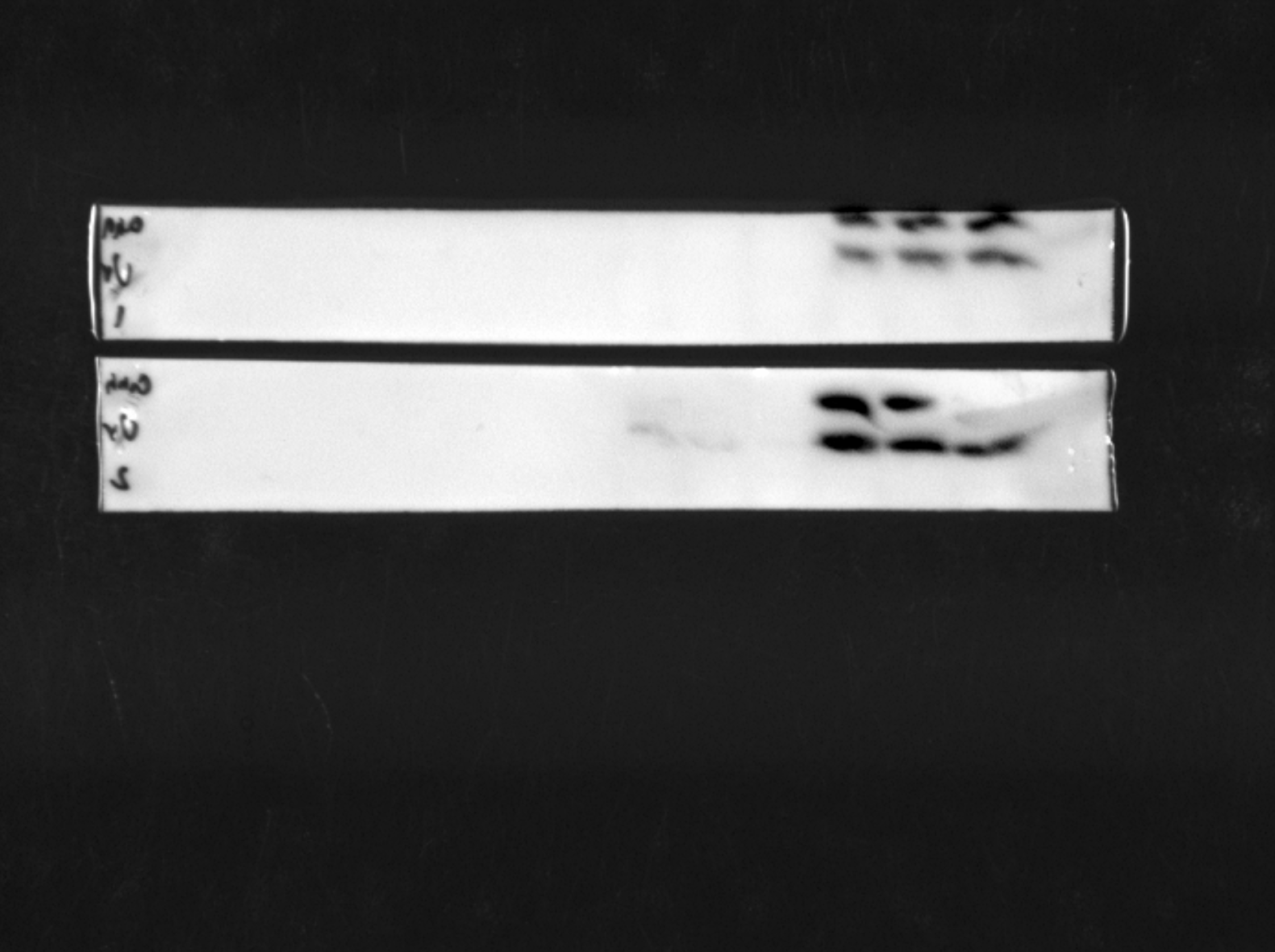

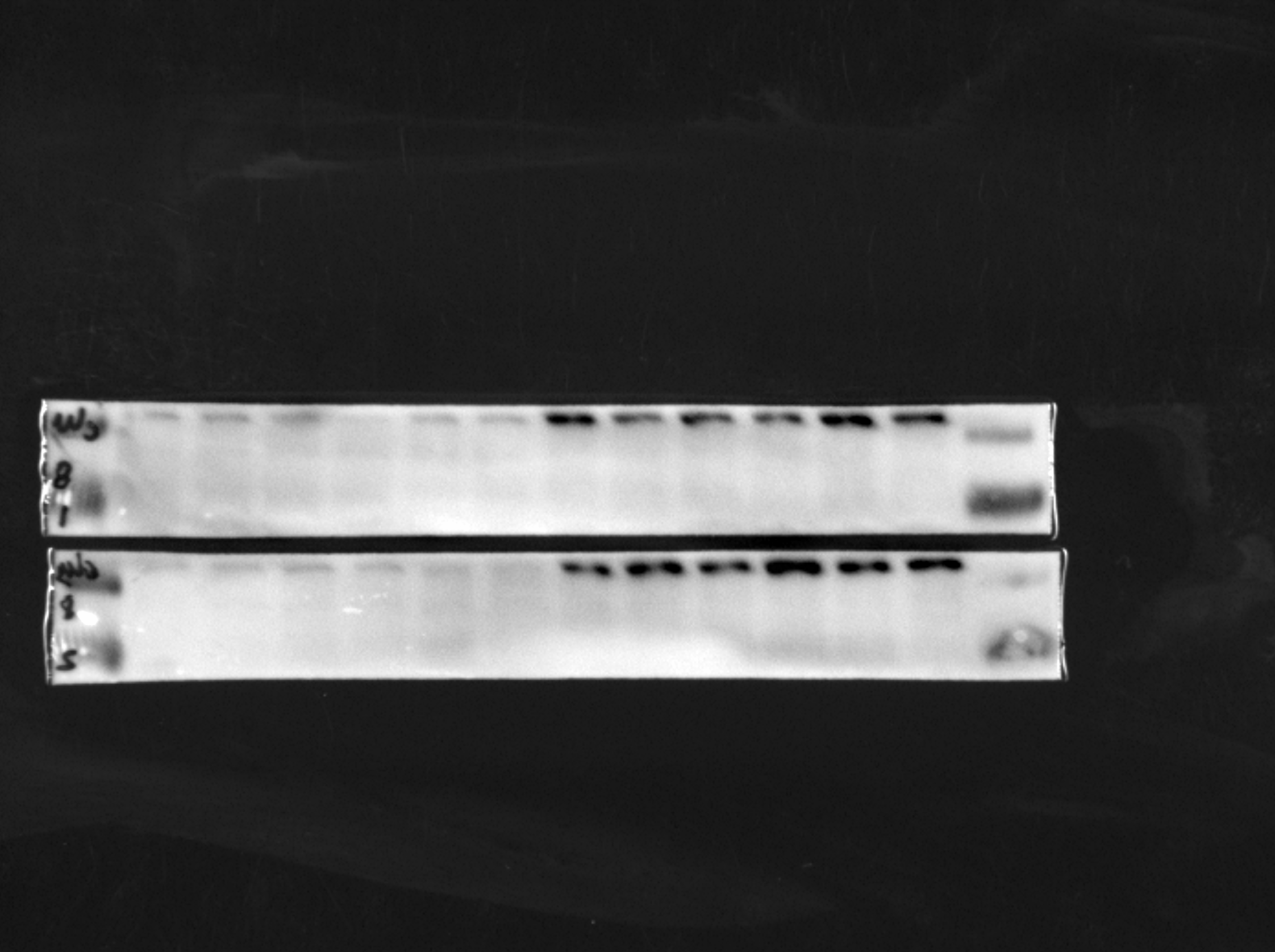

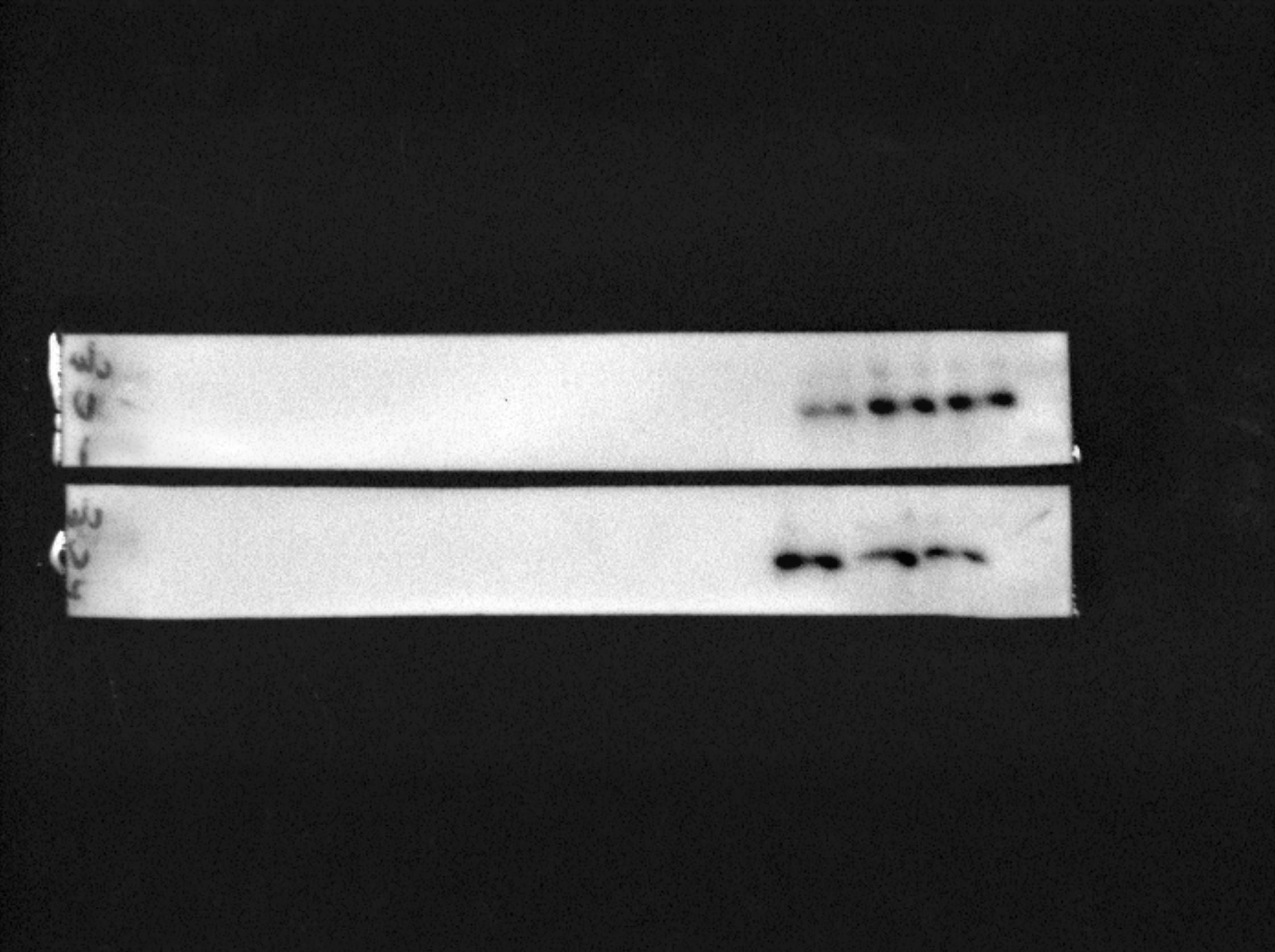

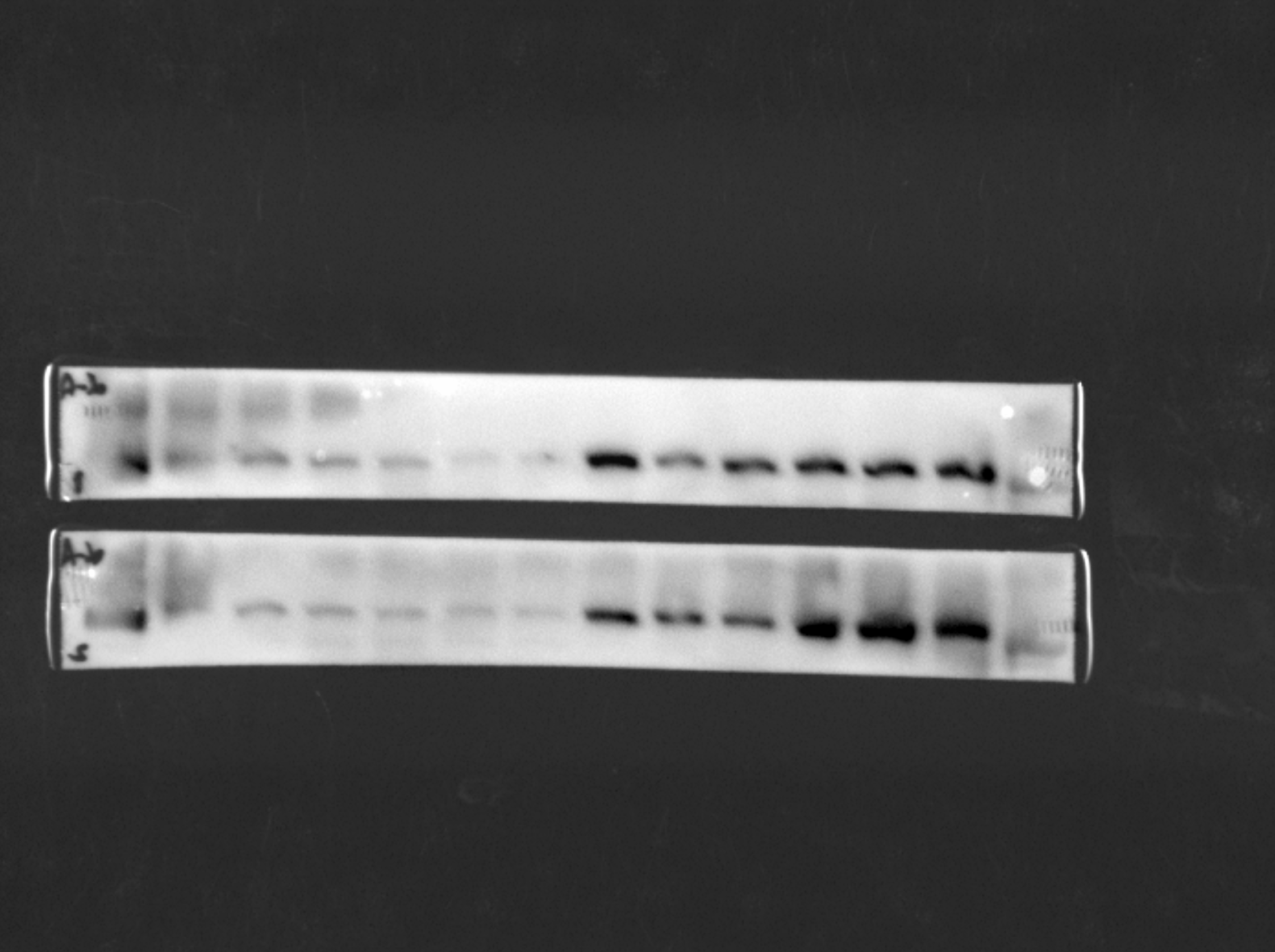

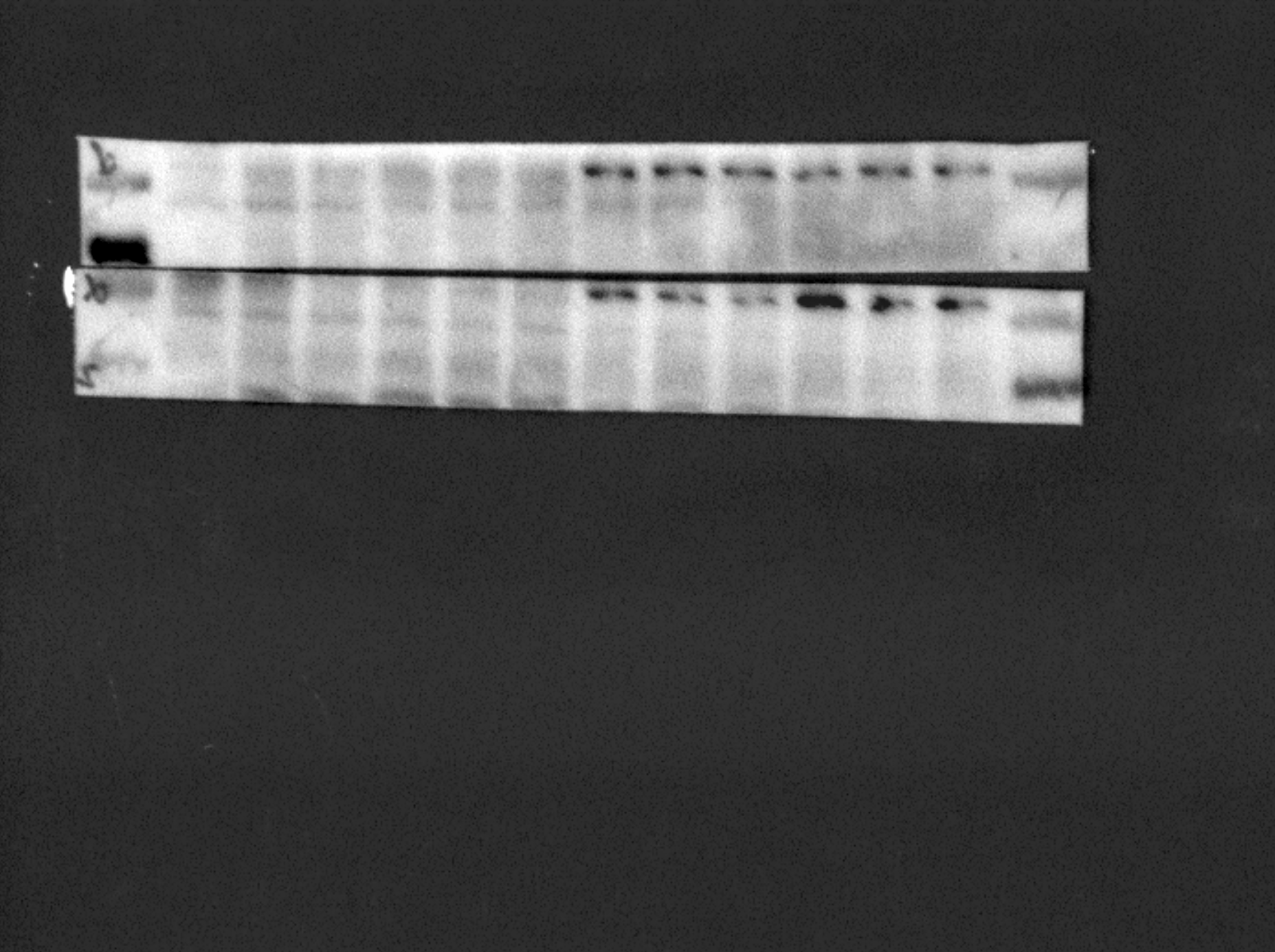

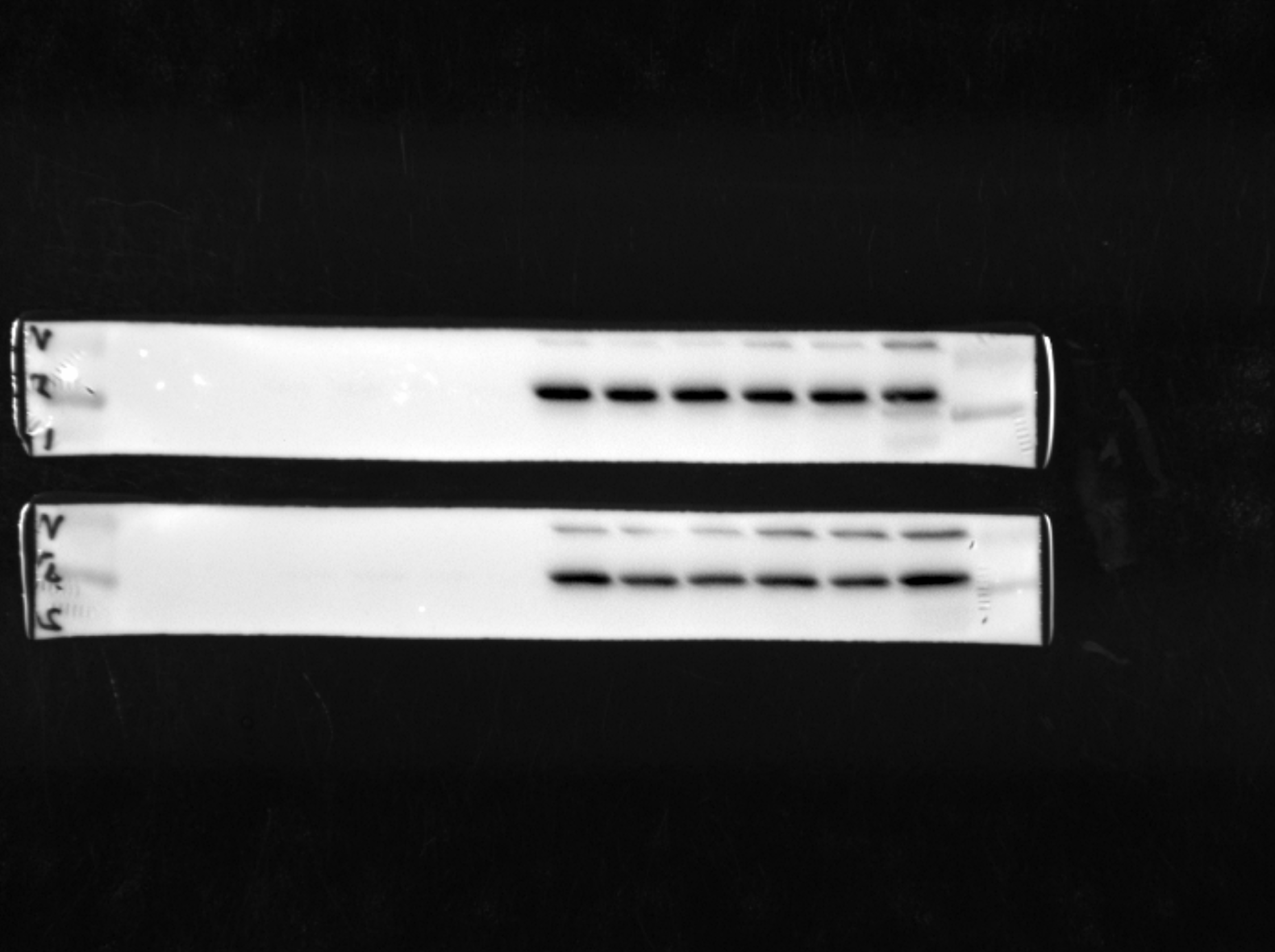

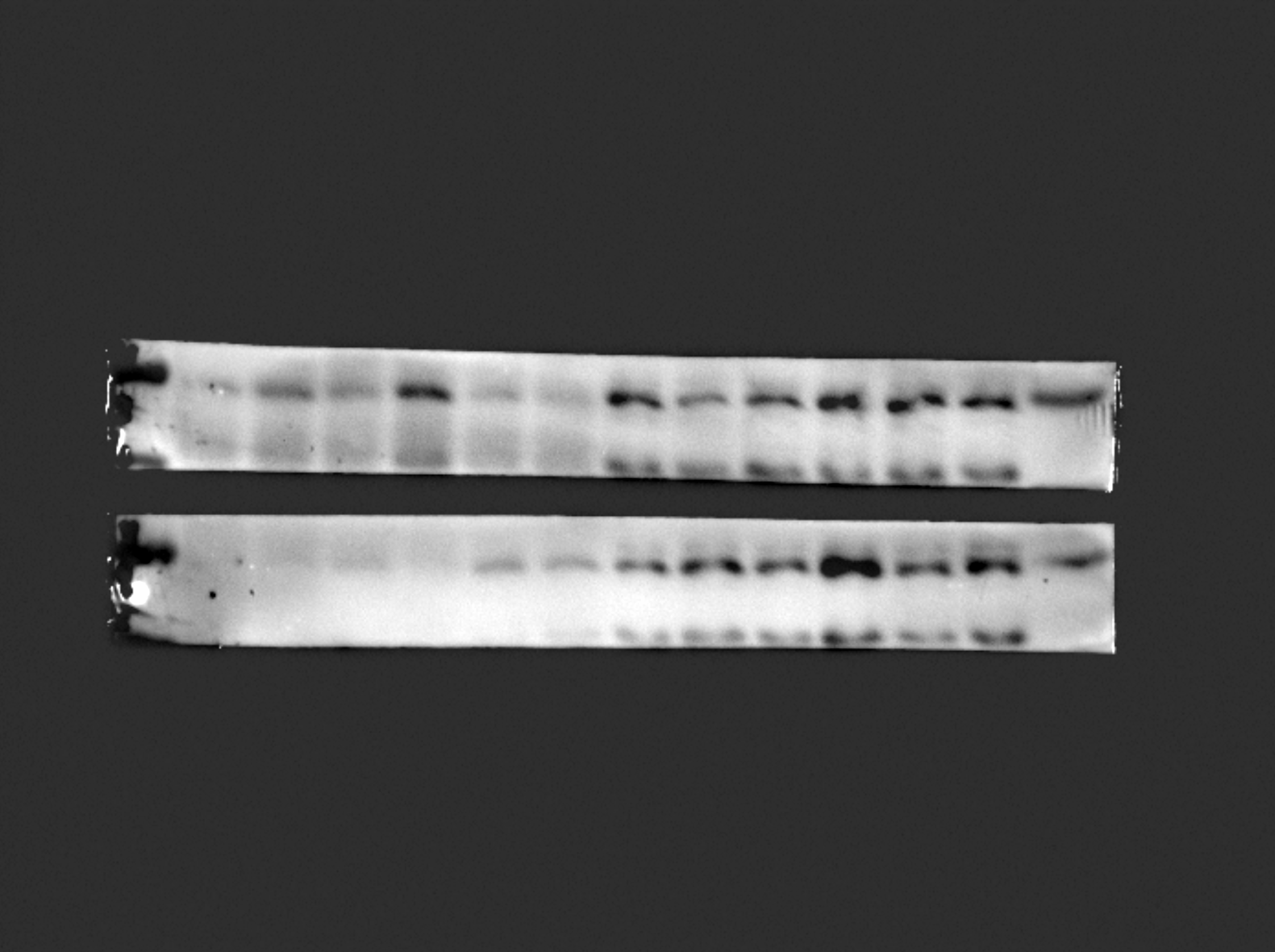
**

40KD-

40KD-

40KD-

**Annexin A1**

**clusterin**

40KD-

100KD-

**Alpha-actinin 1**

100KD-

70KD-

55KD-

**Moesin**

70KD-
